# Effect of dietary canthaxanthin and 25-hydroxycholecalciferol supplementation on the performance of duck breeders under two different vitamin regimens

**DOI:** 10.1186/s40104-016-0062-3

**Published:** 2016-01-22

**Authors:** Zhouzheng Ren, Shizhen Jiang, Qiufeng Zeng, Xuemei Ding, Shiping Bai, Jianping Wang, Yuheng Luo, Zhuowei Su, Yue Xuan, Bing Yao, Fernando Cisneros, Keying Zhang

**Affiliations:** Key Laboratory for Animal Disease-Resistance Nutrition of China Ministry of Education, Institute of Animal Nutrition, Sichuan Agricultural University, Chengdu, 611130 Sichuan China; DSM (China) Ltd., PuDong New Area 201203, Shanghai, P. R. China; DSM Nutritional Products Ltd., Animal Nutrition & Health, Wurmisweg 4303, Kaiseraugst, Switzerland

**Keywords:** Canthaxanthin, Duck breeder, Performance, 25-hydroxycholecalciferol, Vitamin

## Abstract

**Background:**

Dietary canthaxanthin (**CX**), 25-hydroxycholecalciferol (**25-OH-D**_**3**_) and vitamins have been widely reported to be involved in productive and reproductive performance of broiler breeders. However, limited information is available for duck breeders. In this study, a total of 1,560 Cherry Valley SM3 duck breeder females and 312 males were used to assess if the addition of CX and 25-OH-D_3_ could increase the performance of duck breeders under two different dietary vitamin regimens. Four diets were used under a 2 × 2 factorial arrangement with 2 kinds of vitamin premixes (REGULAR and HIGH; HIGH premix had higher levels of all vitamins except K_3_ than REGULAR premix), and with or without the supplementation of the mixture of CX (6 mg/kg) and 25-OH-D_3_ (0.069 mg/kg). The ducks were fed *ad libitum* with pelleted diets based on corn-soybean meal from 38 to 77 wk of age.

**Results:**

HIGH vitamin premix decreased malondialdehyde (**MDA**) level (*P* < 0.001) of egg yolk, increased hatchability of fertile eggs (*P* = 0.029), increased hatchability of total eggs (*P* = 0.029), and decreased serum protein carbonyl level (*P* = 0.037) of breeder males. The mixture of CX and 25-OH-D_3_ increased serum calcium of breeder females (*P* = 0.010), decreased the cracked egg rate (*P* = 0.001), increased the pigmentation of egg yolk (*P* < 0.001) and male bill (*P* < 0.001), and decreased MDA level of egg yolk (*P* < 0.001) and male serum (*P* = 0.034). Interactive effects were observed in cracked egg rate (*P* = 0.038), shell thickness (*P* = 0.011) and serum phosphorus (*P* = 0.026) of breeder females. HIGH vitamin premix together with the mixture of CX and 25-OH-D_3_ decreased cracked egg rate and increased shell thickness of duck breeders. Serum phosphorus was decreased in duck breeder females fed REGULAR vitamin premix without the addition of the CX and 25-OH-D_3_ mixture.

**Conclusions:**

Dietary HIGH vitamin premix increased antioxidant status of eggs and breeder males, and increased hatchability. The mixture of CX and 25-OH-D_3_ enhanced egg shell quality, and promoted pigmentation and antioxidant status of eggs and breeder males.

## Background

Breeder poultry dietary vitamins must be provided in the correct amount and form for optimal animal health and productivity. However, vitamin requirements of duck breeders have been rarely researched in recent decades. Although the vitamin requirements for White Pekin duck breeders were given by NRC (1994) [1], the recommendations for most vitamins were estimated based on values obtained from other ages or species, and have often been criticized to bear little relationship with the levels currently used in the industry. In commercial farms, the duck breeders’ vitamin supplementation is always based on recommendations for broiler breeders, and some of the vitamins are formulated into premix without considering the interaction between vitamins [2, 3]. Unbalanced composition of the premix decreases the utilization efficiency of vitamins and does not show an accurate picture of the productive and reproductive potential of duck breeders [4, 5]. A rational use of dietary vitamin premix might be beneficial for poultry producers.

Canthaxanthin (**CX**, an important carotenoid) and 25-hydroxycholecalciferol (**25-OH-D**_**3**_, one of the vitamin D_3_ metabolites) are also potential dietary factors that could be used to benefit the health and performance of poultry breeders. Canthaxanthin is a powerful immunostimulant [6] and antioxidant [7], and it plays important roles in signaling secondary sexual characteristics in animals [8]. Surai et al. [7] and Zhang et al. [9] reported that CX could modulate the antioxidant status and positively influence the performance of broiler breeders. Rosa et al. [10] confirmed that dietary CX supplementation could increase the fertility and hatchability of broiler breeders. The nutritional role of 25-OH-D_3_ has received considerable attention because of its involvement in calcium-phosphorus metabolism and immune response [11]. The use of 25-OH-D_3_ has been reported to increase egg production, shell quality, hatchability and progeny health of hens [12–15]. Based on these reports, we hypothesized that the diet presently used in the duck breeder industry will be insufficient in CX and 25-OH-D_3_ for productive and reproductive goals.

In the present study, productive, reproductive performance, antioxidant status and serum calcium-phosphorus levels of Cherry Valley duck breeders from 38 to 77 wk were assessed to examine the influence of supplementation of CX and 25-OH-D_3_ under two different dietary vitamin regimens.

## Methods

### Trial design and diets

There were four dietary treatments in a 2 × 2 experimental arrangement with two vitamin premixes (REGULAR and HIGH) and with or without the supplementation of the mixture of CX (6 mg/kg) and 25-OH-D_3_ (0.069 mg/kg). The REGULAR level premix was formulated to simulate a commercial premix used in the duck industry in China; and the HIGH premix was designed according to the recommendations in SM3 Commercial Duck Management Manual [16] which is in good match with the DSM Vitamin Supplementation Guidelines (reported as optimum vitamin nutrition for animals) recommended by DSM Nutritional Products Ltd. [17, 18]. All the vitamins, CX and 25-OH-D_3_ used in the present trial were provided by DSM (China) Ltd. (Chengdu, Sichuan Province, P. R. China). Each treatment contained 3 pens with 130 females and 26 males per pen. In total, 1,560 breeder females and 312 males were fed corn-soybean meal-based pelleted diets (particle size = 4.5 mm) from 38 to 77 wk of age (Table 1).

**Table 1:** Composition and nutrient levels of the basal diet (fed-basis)

Item, %	Amount
Ingredient	
Corn	24.4
Soybean meal, 43 %	22.0
Wheat flour	20.0
Rice bran	15.0
Rice bran meal	2.9
Rapeseed meal	5.0
Meat and bone meal	3.5
Calcium carbonate	6.3
L-Lysine-H_2_SO_4_	0.13
D, L-methionine	0.12
Sodium chloride	0.3
Choline chloride, 50 %	0.1
Premix^a, b^	0.25
Analyzed nutrient content	
CP	20.68
Calcium	3.020
Total phosphorus	0.810
DM	86.73
Calculated nutrient content	
ME, MJ/kg	11.00
Nonphytate phosphorus	0.303
Lysine	1.087
Methionine	0.446
Methionine + Cystine	0.705
Threonine	0.749
Tryptophan	0.263

^a^ Supplied per kilogram of diet: copper, 12 mg; iron, 70 mg; zinc, 80 mg; manganese, 100 mg; selenium, 0.25 mg; iodine, 0.2 mg

^b^ Supplied per kilogram of diet. REGULAR vitamin premix: vitamin A, 10,000 IU; vitamin D_3_, 3,000 IU; vitamin K_3_, 5 mg; vitamin E, 30 mg; vitamin B_1_, 4 mg; vitamin B_2_, 6 mg; vitamin B_6_, 4 mg; vitamin B_12_, 0.015 mg; nicotine acid, 50 mg; pantothenic acid, 15; biotin, 0 mg; folic acid, 1 mg. HIGH vitamin premix: vitamin A, 15,000 IU; vitamin D_3_, 4,000 IU; vitamin K_3_, 5 mg; vitamin E, 100 mg; vitamin B_1_, 5 mg; vitamin B_2_, 16 mg; vitamin B_6_, 5 mg; vitamin B_12_, 0.025 mg; nicotine acid, 60 mg; pantothenic acid, 20; biotin, 0.2 mg; folic acid, 2.5 mg

### Bird care

The trial was conducted at Jinyan breeding farm in Mianzhu, Sichuan Province, P. R. China. Duck breeders were fed on straw litter floors (7 m × 7 m) with an outside area (7 m × 10.5 m) including a swimming pool (1 m × 2 m × 0.4 m in width × length × depth), a drinker and a feeder. There were no other vitamins or drugs used beyond the feed. Feed and water were supplied *ad libitum*, and a 17 light: 7 dark photo-period was used during the trial. Experimental procedures were approved by the Animal Care and Use Committee, Sichuan Agricultural University.

### Laying performance and egg quality

Eggs from each pen were collected every morning, identified and recorded. Setting eggs were stored for a maximum period of 7 d at 15 to 16 °C and 70 to 75 % RH until transferred to the incubator. The laying performance (daily laying rate, daily feed intake, egg weight, total egg mass, feed: egg ratio and cracked eggs) per pen was calculated weekly. A DSM Color Fan™ (DSM Ltd.) was used on the last d of wk 4, 8, 12, 16, 24, 32 and 40 of the trial to measure the bill and shank pigmentation of 6 males per pen. At wk 8, 16, 24, 32 and 40 of the trial, 12 setting eggs per treatment (3 pens of 4 eggs each) produced on the last d were used to determine egg shape index (long axis: short axis, mm: mm), yolk pigmentation [EMT-5200 (Robotmation, Co., Ltd., Tokyo, Japan)], shell thickness [mm, ETG-1061A (Robotmation, Co., Ltd., Tokyo, Japan)], shell strength [kg/cm^2^, EFG-0503 (Robotmation, Co., Ltd., Tokyo, Japan)], shell ratio (shell weight: egg weight, %), albumen height (mm, EMT-5200) and Haugh units [Haugh units = 100 log (H − 1.7 W^0.37^ + 7.57), in which H = height of albumen (mm) and W = egg weight (g)].

### Incubation conditions

At wk 8, 12, 16, 20, 24, 28, 32, 36 and 40 of the trial, 126 setting eggs per pen were randomly chosen to determine the fertility and hatchability. The incubation was conducted with a commercial incubator (Yiai 12096, made in Qingdao, China). The temperature was controlled as 37.8, 37.6 and 37.5 °C during d 1 to 14, 15 to 21 and 22 to 25 of incubation at 60 % humidity. We candled eggs at d 7 of incubation to determine the fertility (expressed as the percentage of fertilized eggs in incubated eggs). At d 26 of incubation, eggs were transferred to a hatcher with 36.5 °C and 70 % RH to complete the incubation process. At d 28, ducklings were removed from the hatcher, then recorded, weighed and their health status was assessed. Ducklings were considered healthy when they were clean and dry, were free of abnormalities, had complete umbilical scarring, and had bright eyes. The hatchability of fertile eggs was expressed as the number of ducklings obtained from every 100 fertilized eggs, the hatchability of setting eggs was expressed as the number of ducklings obtained from every 100 incubated eggs, and the hatchability of total eggs was expressed as the number of ducklings obtained from every 100 eggs laid by duck breeders. Otherwise, the incubation data from each pen throughout the 40 wk trial was summarized, and the following traits were calculated: fertilized eggs per housed female, ducklings per housed female, and healthy ducklings per housed female.

### Serum and yolk analysis

After 40 wk of the laying trial, 9 females (3 females were taken from each of the three pens) and 6 males (2 males were taken from each of the three pens) per treatment were randomly chosen and bled for the determination of antioxidant status. Blood from wing veins was taken by sterilized needles and allowed to clot at room temperature for 2 h before centrifuged at 1,200 × g for 10 min at 4 °C to obtain serum. Serum samples were stored at −20 °C until analyzed for calcium, phosphorus and antioxidant status. In addition, at wk 8, 16, 24, 32 and 40 of the trial, 12 eggs (4 eggs were taken from each of the three pens) per treatment were randomly chosen and the yolk of each egg was separated and stored at −20 °C for analysis. Malondialdehyde (**MDA**) and protein carbonyl were used to evaluate the antioxidant status of serum and yolk.

Serum and yolk were treated with thiobarbituric acid to generate a colored product to measure MDA (as a measure of lipid oxidation) content. Colorimetric method was used to measure the colored product at 532 nm [19].

Serum and yolk protein carbonyl contents were measured using a modification of the method reported by Reznick et al. [20]. Briefly, samples were dissolved using a dinitrophenylhydrazine (DNPH)-HCl solution (blanks were conducted simultaneously by using HCl alone), vortexed for 1 min, then heated in a 37 °C water bath for 30 min in darkness. After the water bath process, proteins were precipitated using trichloroacetic acid and the sediments were washed four times with an absolute ethyl alcohol/ethyl acetate mixture (1:1). Washings were carried out by vortexing of the sediments in the washing solution, and centrifugation at 13,800 × g for 10 min at 4 °C. Finally, the sediments were solubilized in 6 M-guanidine-HCl solution and the absorbance was measured at 370 nm. In addition, total protein content of the samples was measured using a Coomassie Brilliant Blue (**CBB**) method [21] and results were expressed as nanomoles of protein carbonyl in per gram of protein.

Kits for calcium, phosphorus, MDA, protein carbonyl and CBB used in this trial were obtained from Nanjing Jiancheng Bioengineering Institute (Nanjing, Jiangsu Province, P. R. China).

### Statistical analysis

Data were analyzed by ANOVA as a 2 × 2 factorial using GLM procedures of SPSS 17.0 (SPSS Inc., Chicago, IL). The main effects (vitamin premix, mixture of CX and 25-OH-D_3_) and interactions between the two factors were carried out. Duncan’s test was applied when any of the interactions showed significance. Pen was the experimental unit. Data are shown as the LSmeans and pooled SEM. The results were considered significantly different at *P* ≤ 0.05.

## Results

### Laying performance

The laying performance of duck breeders is presented in Table 2. Neither vitamin premix nor the mixture of CX and 25-OH-D_3_ affected daily laying rate, daily feed intake, egg weight, total egg mass and feed: egg ratio of duck breeders (*P* > 0.05). However, the supplementation of the mixture of CX and 25-OH-D_3_ increased the bill pigmentation (*P* < 0.001) and shank pigmentation (*P* < 0.001) of breeder males. Moreover, the cracked egg rate was decreased (*P* = 0.001) by the addition of the mixture of CX and 25-OH-D_3_, and a significant interaction (*P* = 0.038, vitamin premixes × mixture of CX and 25-OH-D_3_) was observed in cracked egg rate which was minimized when breeders were fed with HIGH vitamin premix together with the supplementation of the mixture of CX and 25-OH-D_3_. There were no interactions in other laying traits (*P* > 0.05).

**Table 2:** Laying performance of duck breeders from 38 to 77 wk of age^d^

Vitamin level	CX + 25-OH-D_3_	Daily laying rate, %	Daily feed intake, g	Egg weight, g	Total egg mass, kg	Feed: egg, kg:kg	Cracked eggs, %	Pigmentation of males^e^	
								Bill	Shank
REGULAR	-	82.7	239	92.1	21.3	3.80	1.4^ab^	7.6	10.4
REGULAR	+	81.2	236	91.6	20.8	3.82	1.3^b^	10.7	14.7
HIGH	-	83.9	239	92.1	21.6	3.74	1.5^a^	7.8	10.8
HIGH	+	82.6	237	92.5	21.4	3.74	1.1^c^	11.0	14.9
SEM		0.7	1.0	0.2	0.2	0.03	0.04	0.5	0.6
*P*-value									
Vitamin		0.373	0.831	0.216	0.324	0.297	0.305	0.065	0.062
CX + 25-OH-D_3_		0.366	0.179	0.885	0.409	0.900	0.001	<0.001	<0.001
Vitamin × (CX + 25-OH-D_3_)		0.946	0.737	0.168	0.763	0.821	0.038	0.794	0.419

^a-c^ Different superscripts in a row indicate differ significantly (*P* ≤ 0.05)

^d^ *CX* canthaxanthin, *25-OH-D*_*3*_ 25-hydroxycholecalciferol

^e^ Mean of 7 times of color measure at wk 4, 8, 12, 16, 24, 32 and 40 of the trial

### Egg quality

Supplementation of the mixture of CX and 25-OH-D_3_ significantly increased the yolk pigmentation (*P* < 0.001, Table 3). Interaction between vitamin premix and the CX and 25-OH-D_3_ mixture was found in shell thickness (*P* = 0.011). HIGH vitamin premix together with the mixture of CX and 25-OH-D_3_ significantly increased egg shell thickness.

**Table 3:** Egg quality of duck breeders from 38 to 77 wk of age^c, d^

Vitamin level	CX + 25-OH-D_3_	Egg shape index (long:short)	Yolk pigmentation	Shell thickness, mm	Shell strength, kg/cm^2^	Shell ratio, %	Albumen height, mm	Haugh units
REGULAR	-	1.349	1.9	0.334^ab^	4.00	9.9	8.2	80.6
REGULAR	+	1.341	12.6	0.325^b^	3.97	9.8	8.6	83.0
HIGH	-	1.347	2.1	0.324^b^	4.10	9.9	8.3	81.2
HIGH	+	1.344	12.5	0.342^a^	4.24	10.0	8.7	83.2
SEM		0.004	0.4	0.002	0.07	0.1	0.1	0.8
*P*-value								
Vitamin		0.934	0.914	0.779	0.128	0.436	0.668	0.732
CX + 25-OH-D_3_		0.477	<0.001	0.189	0.758	0.866	0.233	0.264
Vitamin × (CX + 25-OH-D_3_)		0.761	0.382	0.011	0.688	0.412	0.833	0.897

^a, b^ Different superscripts in a row indicate differ significantly (*P* ≤ 0.05)

^c^ Mean of 5 times of sample analysis at wk 8, 16, 24, 32 and 40 of the trial

^d^ *CX* canthaxanthin, *25-OH-D*_*3*_ 25-hydroxycholecalciferol

### Fertility and hatchability

Dietary HIGH vitamin premix increased the hatchability of fertile eggs (*P* = 0.029, Table 4) and the hatchability of total eggs (*P* = 0.029). However, individual or interactive effects of vitamin premix and the CX and 25-OH-D_3_ mixture were not found (*P* > 0.05) in the following traits: fertility, fertilized eggs per housed female, hatchability of setting eggs, ducklings per housed female, healthy ducklings, healthy ducklings per housed female, and 1-d-old weight of ducklings.

**Table 4:** Fertility and hatchability of duck breeders from 38 to 77 wk of age^a, b^

Vitamin level	CX + 25-OH-D_3_	Fertility, %	Fertilized eggs (per housed female)	Hatchability of fertile eggs, %	Hatchability of setting eggs, %	Hatchability of total eggs, %	Ducklings (per housed female)	Healthy duckling, %	Healthy ducklings (per housed female)	1-d-old weight, g
REGULAR	-	87.7	163.7	91.7	80.4	77.4	150.2	90.8	136.3	59.2
REGULAR	+	88.9	161.7	90.9	80.8	77.5	147.1	91.1	134.1	58.8
HIGH	-	87.7	161.3	93.1	81.6	78.4	150.1	90.6	136.0	59.4
HIGH	+	89.6	167.9	92.9	83.1	80.1	155.8	92.7	144.5	59.6
SEM		0.4	2.6	0.4	0.5	0.5	2.6	0.4	2.5	0.2
Vitamin		0.704	0.759	0.029	0.065	0.029	0.475	0.353	0.368	0.332
CX + 25-OH-D_3_		0.106	0.711	0.430	0.256	0.207	0.826	0.147	0.574	0.794
Vitamin × (CX + 25-OH-D_3_)		0.721	0.491	0.657	0.516	0.276	0.470	0.255	0.340	0.592

^a^ Mean of 9 times of incubation at wk 8, 12, 16, 20, 24, 28, 32, 36 and 40 of the trial

^b^ *CX* canthaxanthin, *25-OH-D*_*3*_ 25-hydroxycholecalciferol

### Antioxidant status

HIGH vitamin premix decreased the MDA level of egg yolks (*P* < 0.001) and the serum protein carbonyl level of males (*P* = 0.037, Table 5). The MDA level of egg yolks (*P* = 0.034) and male serum (*P* = 0.034) were decreased with the supplementation of the CX and 25-OH-D_3_ mixture in feed. However, the antioxidant status of breeder females was not affected (*P* > 0.05) by the two experimental factors. No significant interactions were observed in the antioxidant status traits (*P* > 0.05).

**Table 5:** Antioxidant status of duck breeders and eggs^a^

Vitamin level	CX + 25-OH-D_3_	Female		Male		Egg yolk^b^	
		MDA, nmol/mL	Protein carbonyl, nmol/mgpro	MDA, nmol/mL	Protein carbonyl, nmol/mgpro	MDA, nmol/mL	Protein carbonyl, nmol/mgpro
REGULAR	-	10.41	0.95	8.90	1.23	235.81	22.01
REGULAR	+	10.13	0.79	7.71	1.03	181.21	21.51
HIGH	-	10.45	0.80	7.92	0.73	174.69	20.99
HIGH	+	9.27	0.71	7.05	0.91	156.86	18.89
SEM		0.30	0.13	0.25	0.08	5.34	0.65
*P*-value							
Vitamin		0.505	0.665	0.084	0.037	<0.001	0.175
CX + 25-OH-D_3_		0.235	0.641	0.034	0.934	<0.001	0.302
Vitamin × (CX + 25-OH-D_3_)		0.460	0.895	0.729	0.204	0.073	0.516

^a^ *CX* canthaxanthin, *25-OH-D*_*3*_ 25-hydroxycholecalciferol, *MDA* malondialdehyde

^b^ Mean of 5 times of sample analysis at wk 8, 16, 24, 32 and 40 of the trial

### Serum calcium and phosphorus

Serum calcium levels of breeder females were greatly increased (*P* = 0.010, Table 6) by the supplementation of the mixture of CX and 25-OH-D_3_ in diet. Interaction between vitamin premix and the CX and 25-OH-D_3_ mixture was found to influence the serum phosphorus of breeder females (*P* = 0.026). Decreased level of serum phosphorus was observed in duck breeder females under REGULAR vitamin premix without the addition of the CX and 25-OH-D_3_ mixture. There were no differences (*P* > 0.05) between treatments in serum calcium and phosphorus levels of males.

**Table 6:** Serum calcium and phosphorus levels of duck breeders^c^

Vitamin level	CX + 25-OH-D_3_	Female		Male	
		Calcium, mmol/L	Phosphorus, mmol/L	Calcium, mmol/L	Phosphorus, mmol/L
REGULAR	-	2.92	2.70^b^	2.18	1.07
REGULAR	+	3.42	3.67^a^	2.47	1.62
HIGH	-	3.12	3.83^a^	2.42	1.58
HIGH	+	3.25	3.52^a^	2.50	1.63
SEM		0.06	0.15	0.05	0.09
*P*-value					
Vitamin		0.889	0.085	0.208	0.151
CX + 25-OH-D_3_		0.010	0.245	0.077	0.100
Vitamin × (CX + 25-OH-D_3_)		0.125	0.026	0.290	0.172

^a, b^ Different superscripts in a row indicate differ significantly (*P* ≤ 0.05)

^c^ *CX* canthaxanthin, *25-OH-D*_*3*_ 25-hydroxycholecalciferol

## Discussion

Studies of the application of 25-OH-D_3_ in laying hen diets containing higher than NRC (1994) [1] recommended level of vitamin D_3_ remain controversial. Roland and Harms [22] reported that the supplementation of 25-OH-D_3_ (1.1 mg/kg) had no significant effect on the laying performance of hens fed a basal diet containing 2200 IU/kg vitamin D_3_. However, Torres et al. [23] found that the supplementation of 25-OH-D_3_ (0.035 or 0.069 mg/kg) in diets containing 2000 IU/kg vitamin D_3_ resulted in increased egg shell quality. In the study of Zang et al. [24], hens fed a diet containing 2500 IU/kg vitamin D_3_ and 0.035 mg/kg 25-OH-D_3_ had a reduced number of cracked eggs when compared with a diet containing 2400 IU/kg vitamin D_3_ without 25-OH-D_3_. Similarly, in this study, dietary supplementation of the CX and 25-OH-D_3_ mixture made no effect on egg production; but decreased cracked egg percent of duck breeders, even both the Regular and the High vitamin premixes had higher level of vitamin D_3_ than NRC (1994) [1] recommendation. In these studies, different responses to 25-OH-D_3_ supplementation were observed when the desired criteria were changed. Response in egg shell quality occurred with 25-OH-D_3_ supplementation above the requirement for egg production. Interestingly, lowest cracked egg rate and highest shell thickness were achieved in the highest total vitamin D supplementation group (HIGH premix together with the CX and 25-OH-D_3_ mixture). Based on these data, we speculate that a re-evaluation of the NRC (1994) [1] vitamin D_3_ recommendation (900 IU/kg) for duck breeders might be needed when using egg shell quality as the parameter. In this study, the supplementation of the mixture of CX and 25-OH-D_3_ also increased female serum calcium level, which may partially account for the increased shell quality [25]. In REGULAR vitamin premix groups, the increased female serum phosphorus induced by CX and 25-OH-D_3_ supplementation may be explained by increased intestinal phosphorus absorption because of increased 1, 25 dihydroxyvitamin D_3_ synthesis [26].

Higher than NRC (1994) [1] levels of vitamins have many times been reported to reveal no beneficial effects on egg production of laying hens [15, 22, 24]. However, hens revealed to have greater vitamin requirements for hatchability than for laying, as increased hatchability was achieved by maternal supplementation of high levels of vitamins [27, 28]. Similarly, in the current study, maternal HIGH vitamin premix made no effects on egg production, but increased the hatchability of fertile eggs and the hatchability of total eggs. In the avian system, the embryo develops outside the maternal body, and all the nutrients required by the embryo are pre-deposited inside the egg during egg formation. A balanced nutritional status of breeder eggs is essential for the development of embryo [29, 30]. In the current study, the increased levels of vitamins in HIGH premix may have helped to modify the vitamin composition of duck breeder eggs [7, 24, 31], and benefit the embryo development. Egg production has long been used as the key criteria to evaluate the vitamin requirement of hens. However, parameters for assessing needs are now more complex as more and more focus goes to the reproductive efficiency and health of offspring [15, 28, 32]. Information on vitamin requirements of duck breeders is surprisingly lacking in recent years. Our data indicates that the current REGULAR vitamin premix provides enough vitamins for the laying performance of duck breeders; however, more vitamins might be required for breeding purposes (e.g. hatchability). In addition, the vitamin levels in the current HIGH vitamin premix are in accordance with the commercial recommendations for broiler breeders [17, 33]. Thus, our results indicate that duck breeders probably have similar vitamin requirements as broiler breeders for reproductive performance.

Many vitamins (e.g., vitamin A, C, E, and B1) and pigments (e.g., carotenoids) have been reported to exert antioxidant activity [9, 10, 28, 34]. In this study, either HIGH vitamin premix or the supplementation of the mixture of CX and 25-OH-D_3_ decreased the level of MDA, a lipid peroxidation product, in breeder egg yolk. Chicken embryo is known to undergo reactive oxygen species (**ROS**) formation and lipid peroxidation during the incubation period due to its high polyunsaturated fatty acid contents [35]. The decreased MDA level in egg yolk may further help to reduce the lipid peroxidation and increase the health status of the developing embryo [36]. These data also offer an approach to reduce the economic loss caused by high temperature stress or long-term breeder egg storage, which may increase lipid peroxidation status of breeder eggs and reduce hatchability [33, 37]. The role of CX in yolk pigmentation has been well characterized [38]. Similarly, in the current study, yolk pigmentation was increased by the addition of the mixture of CX and 25-OH-D_3_.

In the present study, the antioxidant and pigmentation status of duck breeder males showed the same trend like the egg yolks as affected by ether HIGH vitamin premix or the supplementation of the CX and 25-OH-D_3_ mixture. In male animals, it is well established that an enhanced antioxidant status correlates with increased semen quality [39]. The current results indicate a potential role of dietary antioxidants in improving the reproductive performance of duck breeder males. The increased pigmentation status of males in the CX and 25-OH-D_3_ mixture supplemented groups might play important roles in the expression of secondary sexual characteristics [8, 40] and worth further investigation. Interestingly, no effects were observed in antioxidant and pigmentation (data not shown) status of duck breeder females. It is noteworthy that maternal dietary antioxidants and pigments could be effectively transferred to the egg yolk, subsequently absorbed into the developing embryo, and distributed in the progeny tissues [7]. In this regard, the increased dietary vitamins and the CX and 25-OH-D_3_ mixture were more likely to be transferred to breeder eggs but not deposited in tissues of breeder females. This is highly consistent with the current observations on antioxidant and pigmentation status of breeder eggs, and suggest the possible use of maternal antioxidants and pigments to promote the quality of newly hatched ducklings.

## Conclusions

HIGH vitamin premix made no effect on egg production and egg quality, but enhanced the antioxidant status of eggs and breeder males, and increased hatchability. The supplementation of the mixture of CX and 25-OH-D_3_ increased egg shell quality and increased the pigmentation and antioxidant status of eggs and breeder males.

## Abbreviations

25-OH-D_3_: 25-hydroxycholecalciferol
ANOVA: analysis of variance
CBB: Coomassie brilliant blue
CX: Canthaxanthin
d: day
DNPH: dinitrophenylhydrazine
GLM: general linear model
LSmeans: least-square means
MDA: malondialdehyde
NRC: National Research Council
ROS: reactive oxygen species
SEM: standard error of measurement
wk: week
